# Modulating Cancer Stem Cell Characteristics in CD133+ Melanoma Cells through Hif1α, KLF4, and SHH Silencing

**DOI:** 10.1021/acsomega.5c00799

**Published:** 2025-04-18

**Authors:** Berrin Ozdil, Cigir Biray Avci, Duygu Calik-Kocaturk, Volkan Gorgulu, Aysegul Uysal, Günnur Güler, Nefise Ülkü Karabay Yavaşoğlu, Huseyin Aktug

**Affiliations:** 1 Department of Histology and Embryology, Faculty of Medicine, Suleyman Demirel University, Isparta 32260, Turkey; 2 Department of Histology and Embryology, Faculty of Medicine, Ege University, Izmir 35100, Turkey; 3 Department of Physics, Biophysics Laboratory, Izmir Institute of Technology, Izmir 35430, Turkey; 4 Department of Medical Biology, Faculty of Medicine, Ege University, Izmir 35100, Turkey; 5 Dr. İsmail Fehmi Cumalioglu City Hospital, Tekirdağ 59030, Turkey; 6 Department of Biology, Faculty of Science, Ege University, Izmir 35100, Turkey

## Abstract

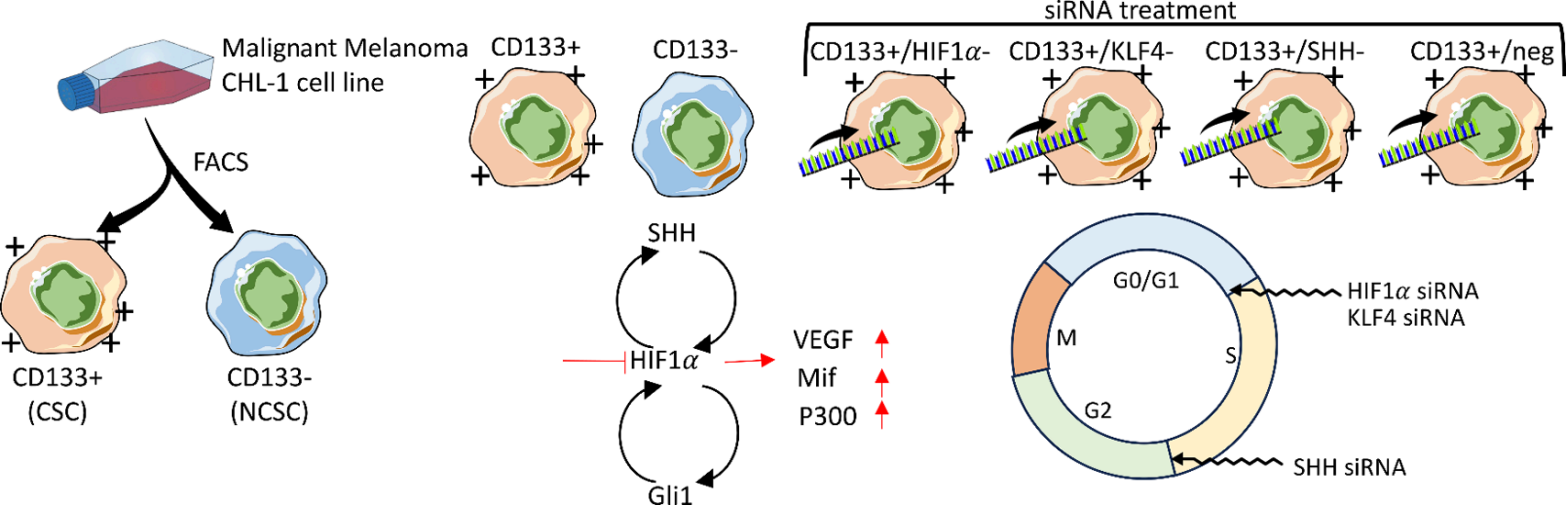

Malignant melanoma is a highly aggressive form of skin cancer, partly driven by a subset of cancer stem cells (CSCs) with remarkable capacities for self-renewal, differentiation, and resistance to therapy. In this study, we examined how silencing three key genes—Hif1α, KLF4, and SHH—affects CSC characteristics. Using small interfering RNA (siRNA)-based approaches, we observed significant changes at both the gene and protein levels, shedding light on how these pathways influence melanoma progression. Our results demonstrated that silencing these genes reduces the stem-like features of CSCs. Notably, Hif1α silencing triggered a marked decrease in hypoxia-related gene expression, while targeting SHH led to a reduction in Gli1, a downstream effector of SHH signaling, highlighting its potential as a therapeutic target. We also observed changes in epigenetic markers such as HDAC9 and EP300, which play crucial roles in maintaining stemness and regulating gene expression. Interestingly, these interventions appeared to reprogram CSCs, pushing them toward a phenotype distinct from both traditional CSCs and non-stem cancer cells (NCSCs). Our findings emphasize the importance of targeting key signaling pathways in melanoma CSCs and underscore the value of mimicking the tumor microenvironment in experimental models. By revealing the dynamic plasticity of melanoma CSCs, this study offers fresh insights into potential therapeutic strategies, particularly using siRNA to modulate pathways associated with tumor progression and stem cell behavior.

## Highlights

- Distinct roles of Hif1α, KLF4, and SHH in melanoma CSC modulation are identified.
- SHH pathway proteins Gli1 and PTCH2 emerge as potential therapeutic targets.
- The siRNA-based approach reveals dynamic CSC reprogramming and subtype generation.

## 1 Introduction

Malignant melanoma, a highly aggressive skin tumor originated from melanocytes, displays a complex genetic profile influenced by both genetic and environmental factors.^1^ The abundance and activity of CSCs are associated with the aggressiveness of malignant melanoma as in other cancer types.^2^ The detection of tumor heterogeneity, undifferentiated molecular markers, and increased tumor identity of melanoma subtypes with embryonic-like developmental plasticity strongly suggests the presence and involvement of malignant melanoma stem cells in the initiation and progression of this malignancy.^3^

Most physiological functions in cellular processes are regulated by the oxygen concentration. Cancer cells can adapt to low oxygen levels by regulating the hypoxia signaling, where the key regulator is Hif1α.^4,5^ In cancer progression, hypoxia, which activates angiogenesis, thereby increases the risk of invasion and metastasis.^5,6^ This increases cell survival within the tumor as well as suppresses antitumor immunity and inhibits the therapeutic response. A study conducted on hypoxia in the GBM cell line demonstrated that the upregulation of Hif1 and Hif2 resulted in elevated levels of SOX2 and KLF4, thereby affecting the stemness and cell cycle of the cells through this signaling pathway.^7^

Supporting the hypoxia mechanism, here, we focus on KLF4 and SHH signaling for the changes in stemness regulators. The role of KLF4 in melanoma remains unknown despite its strong expression in postmitotic epidermal cells and terminally differentiated skin and gut cells.^8^ A 2017 study investigating KLF4’s function in melanoma cell lines (SK-Mel-2, SK-Mel-5, SK-Mel-28, SSM2c, M26c, M33x, and M51) and its interaction with the MAPK signaling system found strong expression of KLF4 in human melanomas. In addition, knockdown of KLF4 decreased patient melanoma cell proliferation and promoted cell death, while ectopic expression of KLF4 increased melanoma cell growth by lowering apoptosis.^8^ Although KLF4 has been extensively studied in melanoma, the research on melanoma CSCs remains limited. Understanding the role of KLF4 protein is crucial for studying and characterizing CSCs.

The SHH protein, a key player in the Hedgehog signaling pathway, contributes significantly to various developmental processes, especially in nervous system development, where it acts as a morphogen during the early developmental stages. Besides its crucial role in embryonic development, Hedgehog signaling has been shown to be involved in vasculogenesis and angiogenesis.^9^ Despite its prominence in the developmental process, the SHH signaling pathway is also actively involved in studies on CSC. The hypoxia-induced activation of the SHH signaling pathway in different tissues^10^ highlights its importance in CSC biology.^11^ However, research on SHH signaling in melanoma cell lines is limited, with most studies focusing on PI3K rather than Gli1.^12,13^ In a thesis study that has not yet been published, CHL-1 melanoma cells were transfected with a malignant vector carrying the SHH protein, and the subsequent expression of Gli1/Gli3 and activation of the PI3K signaling pathway were examined.^14^ Here, SHH gene expression was directed using siRNA technology, and cells were classified as either CSCs or NCSCs.

The tumor microenvironment is another factor affecting tumor character; extracellular matrix molecules are responsible for tumor progression. Therefore, it is extremely important to establish the tumor microenvironment to create specific target therapies. To increase the metastatic capacity, melanoma cells manipulate the extracellular matrix and secrete extracellular factors for this,^15^ and each change or modification on the extracellular matrix is effective on the cancer biological behavior and has to be examined.^16−18^

The main aim in this study is to demonstrate the differential signaling in CSC and NCSC in the presence of an extracellular matrix component, Matrigel, with or without silencing the stem cell character by targeting the Hif1α, KLF4, and SHH genes in CSCs. Silencing these genes altered the cell characteristics, resulting in new phenotypes.

## 2 Methods

### 2.1 Cell Lines and Culture

The nonpigmented human melanoma cell line CHL-1 (ATCC CRL-9446) was cultured under normoxic conditions in EMEM (Eagle’s Minimum Essential Medium) (Biowest L0416) supplemented with 10% fetal bovine serum (Biowest, S1810) at 5% CO_2_ at 37 °C. Regular authentication and mycoplasma infection checks were conducted on the cell line. For experimental consistency and reproducibility, cells within passages 6–8 were selected for flow cytometry sorting.

### 2.2 Fluorescence-Activated Cell Sorting (FACS)

The cells were detached from the surface of the flask by trypsinization, which is a standard method for enzymatically releasing adherent cells. Following detachment, the cells were washed twice with cold 1× PBS to remove any residual trypsin and cellular debris. The collected cells were then diluted to a concentration of 10^6^ cell/ml in 10 mL cold 1× PBS. Following this, they were incubated with 10 μL CD133 phycoerythrin (PE)-labeled antibody (Miltenyi Biotec Ltd. 130-113-186) and 10 μL DAPI for 15 min at +4 °C. After the incubation period, the cells were washed with 1× PBS supplemented with 1% dialyzed fetal bovine serum (FBS). Control cells were subjected to staining with DAPI only without the addition of any antibodies. Subsequently, cell sorting was performed using a BD FACS Diva 8.0. The collection tubes labeled CD133+ were designated as CSCs, while CD133– cells were categorized as NCSCs.

Within the malignant melanoma cell population, a CD133+ cell subset ranged from 0.1 to 0.4%. The CD133+ cell subset was used as passage 2 to passage 4 and utilized in subsequent experimental procedures (see Figure S1A). A sphere formation assay was performed as a functional assay to confirm the stemming property of CD133+ cells. To induce spheroid formation, we prepared a surface coated with agarose. Two milliliters of 1% agarose (cat. no. DF0812-07-1, Difco Laboratories, Inc.; BD Diagnostic Systems, Detroit, MI, USA) in EMEM without FBS was poured into each well of a six-well plate and allowed to solidify. Once polymerization was complete, 1 × 10^5^ cells were seeded per well and incubated at 37 °C with 95% air and 5% CO_2_. Fresh medium was gently added at 3 days, and spheroid formation was documented by photographing the cells on day 5^19^ (Figure S1B). Cell counting was conducted after sorting and experimental procedures using a Muse Cell Analyzer, a specialized instrument for automated cell counting and analysis.

### 2.3 Matrigel Coating

To better mimic the tumor microenvironment, the CSCs were cultured in the presence of extracellular matrix components. Before the cells were seeded, the surface was coated with Matrigel (Corning Matrigel Basement Membrane Matrix, 354234). To ensure optimal coating, all materials required for this process were precooled to +4 °C and handled accordingly. The Matrigel used for surface coating was prepared by mixing it with a serum-free medium at a ratio of 1:4 while it was in liquid form, ensuring that no bubbles formed. On a 15 mm coverslip (with a surface area of 1.77 cm^2^), 100 μL of the Matrigel solution was applied, while on the surface of a six-well plate (with a surface area of 9.6 cm^2^), 542 μL of the Matrigel solution was distributed on ice. Coverslips measuring 15 mm were utilized for immunofluorescence, atomic force microscopy (AFM), scanning electron microscopy (SEM), and X-ray photoelectron spectroscopy (XPS) analysis, while the six-well plates were used for reverse transcription-polymerase chain reaction (RT-PCR). The Matrigel, which remained in the state at +4 °C, was uniformly distributed across the surface during Matrigel coating. To achieve even distribution, the surface containing the liquid Matrigel and in contact with a cold surface was placed on a shaker. Following the coating of Matrigel, the surface was then transferred to a standard cell culture incubator set at 37 °C. Through observation, it was determined that a 20 min incubation period was optimal for the complete polymerization of the Matrigel coating, ensuring a stable and supportive environment for subsequent cell seeding. The surface coating of Matrigel was further characterized using atomic force microscopy (Bruker Dimension Edge with ScanAsyst AFM (Bruker, Germany))^20,21^ and scanning electron microscopy (Thermo Scientific Apreo S LoVac SEM (ThermoFisher Scientific, US))^21,22^ imaging techniques (Figure S1C).

### 2.4 Small Interfering RNA (siRNA) Transfection

To silence the Hif1α, KLF4, and SHH genes, CD133+ malignant melanoma CSCs were transfected with 0–200 nM siRNA (On-Targetplus Human siRNA, Smartpool, L-005089-00-0005, L-004018-00-0005, and L-006036-00-0005 Horizon). The transfection was performed to determine the optimal siRNA dosage, and gene expression levels were subsequently assessed using RT-PCR for the control. The fold-change cutoff limit was set at 2, with changes between 0 and 2 considered insignificant. In the experiment, siRNA concentrations ranging from 0 to 200 nM were used. After siRNA treatments at varying dosages, the cells underwent RT-PCR analysis. Specifically, the use of 5 nM Hif1α siRNA resulted in a fold change of −56.44×. For KLF4, the highest fold change (−4.56× fold) was observed at 5 nM, while SHH gene silencing was achieved at 25 nM (−3.84× fold). Subsequently, 5 nM Hif1α and KLF4 and 25 nM negative control siRNA and SHH were used for further experiments. Cells were fixed after 24 h of incubation following siRNA transfection for analysis.

### 2.5 Reverse Transcription-Polymerase Chain Reaction (RT-PCR)

Cells were incubated for 24 h following siRNA transfection after seeding a concentration at 10^5^ cells/mL. The cells were rinsed with 1× phosphate-buffered saline (PBS), detached from the surface with the StemPro Accutase Cell Dissociation Reagent (Thermo Fisher), and dissolved in the RNA buffer (Roche). The subsequent steps were conducted in accordance with the Roche isolation kit. RT-PCR was conducted on the LightCycler 2.0 system (Roche, Basel, Switzerland) using 50 ng of template cDNA and FastStart Universal SYBR Green Master Mix (Roche Hellas). Roche RealTime ready custom panel 96–96+ (reference no. 05582563001; lot no. 0000034886, Germany) was used for the gene expression profiling. PCR quality and primer specificity were verified through melting curve analysis, and relative gene expression was calculated using the ΔΔCt method. Sampling was performed three times. The heatmap with hierarchical trees was generated using Clustvis.^23^

### 2.6 Immunofluorescence Staining

After 24 h of siRNA transfection, cells were fixed using 4% paraformaldehyde (PFA) (Sigma P-6148) and permeabilized with 0.25% TritonX-100 (Biotech, C34H62O11). Subsequently, cells were subjected to the blocking with 3% bovine serum albumin (Chem Cruz, sc-2323) to minimize nonspecific binding. Following blocking, cells were incubated overnight at +4 °C with primary antibodies (Hif1α, Bioss bs-0737R; KLF4, Thermo PA1-095; SHH, Santa Cruz, sc-373779; Gli1, Bioss AI05040; Smo, Bioss AA062588; VEGF, bs-1665R; MIF, Santa Cruz sc-271631; MMP9, Bioss bs-4593R; P300, Bioss, bs-5339R; and HDAC9, Bioss bsm-54186R) diluted 1:200 in 1% BSA. Following primary antibody incubation, cells were washed and incubated with secondary antibodies (Alexa Fluor 488-AffiniPure Goat Anti-Rabbit Jackson Immuno Research, 111-545-003) at room temperature for 1 h. Finally, cells were mounted with a Fluoroshield Mounting Medium with DAPI (abcam, ab104139) for nuclear counterstaining. Microscopic images were acquired by using an Olympus BX-51 microscope (Olympus Optical Co., Tokyo, Japan). At least 100 cells were examined for immunofluorescence staining, with comparisons in cell area entirely based on the fluorescence intensity. The protein expression of the cells was statistically compared based on the intensity signals. Hif1α, SHH, Gli, Smo, KLF4, VEGF, MMP9, P300, HDAC9, and MIF proteins were monitored and evaluated in the experimental groups as part of this study.

### 2.7 Enzyme-Linked Immunosorbent Assay (ELISA)

The results obtained from RT-PCR analysis identified Gli1, HDAC9, and MMP9 as the most significantly differentially expressed genes. To further investigate their potential role in paracrine signaling, an enzyme-linked immunosorbent assay (ELISA) was performed. Prior to the assay, all reagents were equilibrated at room temperature before use. The supernatants collected from the cells were processed following the ELISA kit protocol (BT-Lab E6417Hu, E5411Hu, and E0936Hu), as previously described in our earlier study.^24^ The amount of secreted protein was quantified by using a microplate reader set to measure absorbance at 450 nm.

### 2.8 Cell Cycle Analysis

The cell cycle phases were assessed using a Muse Cell Analyzer.^20^ The cells were subjected to a standard passage protocol, cells were harvested, and the cell pellet was collected. The cells were then fixed in 70% ice-cold ethanol and stored at −20 °C overnight to ensure proper fixation. The following day, ethanol was carefully removed from the samples, and cell cycle kit solution (Millipore lot: 2941162) was added to each sample. The samples were incubated in the dark for 30 min. The Muse Cell Analyzer was used to read the samples in the cell cycle analysis mode.

### 2.9 Statistical Analysis

The “Multiple Plate Analysis” tool was used to examine gene expression profiles. For relative quantification, the expression of reference housekeeping genes (ACTB and GAPDH) was measured. The fold change was estimated by using 2^–ΔΔCt^. The experimental groups were compared using the Student *t* test statistical analysis. For all RT-PCR, protein intensity, image processing, and analyses were carried out entirely blinded. Cell cycle analysis was evaluated by IBM SPSS Statistics 25.0. ImageJ/Fiji was used to measure the intensity of proteins (Image analysis software, National Institutes of Health, Bethesda, MD),^25^ with a minimum of 100 cells analyzed from at least 10 photographs obtained from at least three different experiments. The RGB images obtained as raw data were saved as 8-bit black and white images in the channel containing the protein image using the split channels function before the analysis stage. The boundaries of the cells were drawn using the “freehand selection” tool, and the intensities from this area were statistically compared between groups. The intensity values of the groups were normalized using the corrected total cell fluorescence (CTCF) method.^26,27^ The normality of the data was assessed using the Shapiro-Wilk test, while the homogeneity of variance was evaluated through Levene’s test. Except for the RT-PCR data, ANOVA and Bonferroni post hoc test were used to analyze samples with normal distribution, and Kruskal–Wallis and pairwise comparison tests were used to examine samples without normal distribution. Unless otherwise specified, results are presented as mean ± standard deviation (SD). Statistically significant difference was defined as *p < 0.05, **p < 0.01, and ***p < 0.001.

## 3 Results

### 3.1 Gene Expression Profiling and siRNA Modulation in Malignant Melanoma

**Figure 1:**
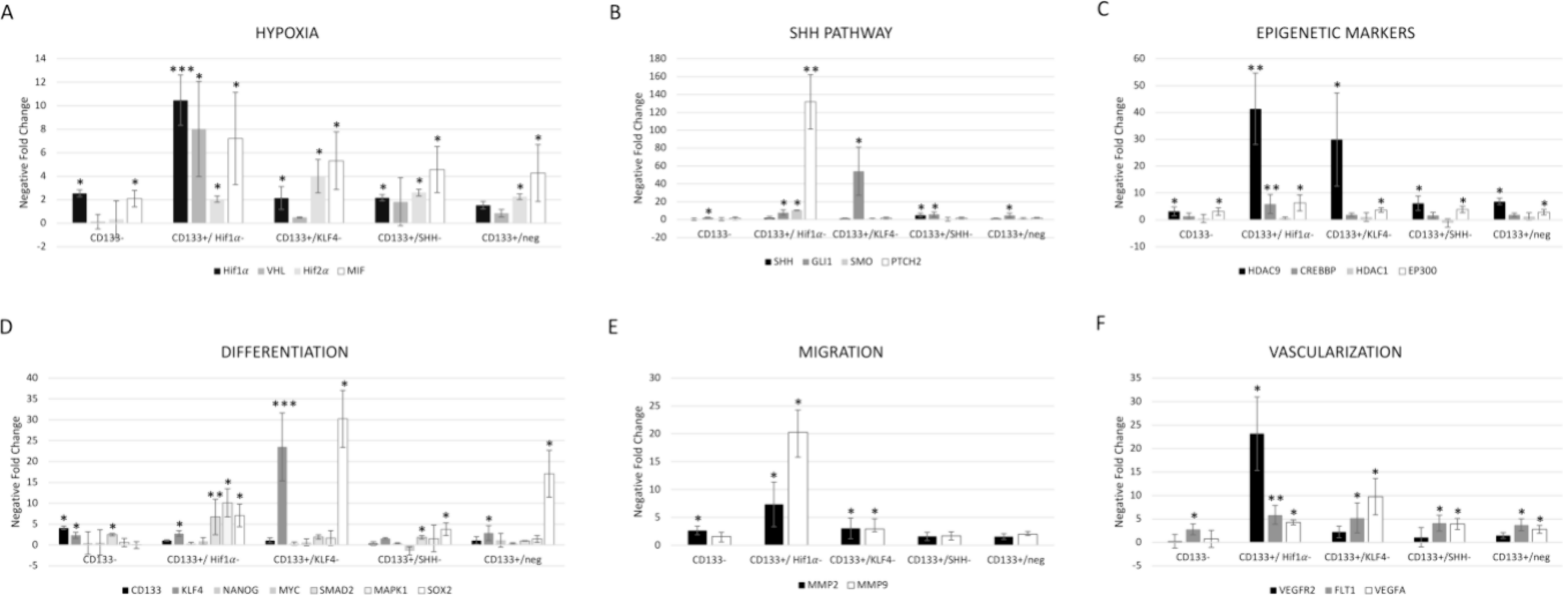
Gene expression profile and differential regulation in cell groups. RT-PCR results normalized with ACTB and GAPDH housekeeping gene expressions. Experimental cell groups’ relative fold changes were calculated with 2^–Avg.(ΔCt)^ formula. The groups were subjected to Student's *t* test and compared with CD133+ cell groups. For improved visualization, negative fold changes were represented as positive values in the graph. Statistically significant differences were indicated as *p < 0.05, **p < 0.01, and ***p < 0.001.

We analyzed the expression levels of 27 genes, including four housekeeping genes, for comprehensive profiling (Figure 1, Figure S2). Gene expression analysis was centered on the CD133+ group, with a 2-fold difference in expression considered significant. Here, we examined genes associated with hypoxia, the SHH pathway, epigenetics, cellular differentiation, migration, and vascularization signaling pathways.

#### 3.1.1 Comparative Analysis of CSCs and NCSCs at the Transcription Level

A comparison between CSCs and NCSCs is crucial for addressing the critiques on signaling pathways. The gene expression comparison between CSC and NCSC populations displayed significant differences across multiple pathways, highlighting increased expression in several key genes within the CD133+ group. These elevated levels were associated with hypoxia response, SHH signaling, epigenetic markers, stemness, and migration, suggesting that the CSCs may have enhanced pathway activities that support their stemness, tumorigenic potential, and survival in the tumor microenvironment. In this comparison, CD133+ and CD133– cells exhibited distinct patterns in hypoxia related genes Hif1α and MIF. The levels of Hif1α and MIF gene expressions were reported relatively lower in the NCSC group compared to CSC (*p* < 0.05). Another distinctly regulated gene was Gli1 (*p* < 0.05), which is the key molecule in the SHH signaling pathway that plays significant roles in cancer and stem cell biology.^28,29^ Moreover, transcripts of epigenetic markers such as HDAC9 and EP300 were found to be elevated in CSCs compared to NCSCs, suggesting the involvement of epigenetic mechanisms in the resistance of CSCs (*p* < 0.05). Markers related to differentiation (CD133, KLF4, and SMAD2) and migration (MMP2) were more abundant in CSCs compared to NCSCs (*p* < 0.05). It was observed that VEGFR2 and VEGFA gene expression levels were comparable between CD133+ and CD133– cell groups, while FLT1 gene expression was found to be negatively regulated in the CD133– cell group (*p* < 0.05) (Figure 1, Figure S2).

#### 3.1.2 Gene Silencing Resulted in a Decrease in Hypoxia-Related Genes at the RNA Level

The hypoxia-related genes Hif1α, VHL, Hif2α, and MIF displayed significant expression differences across the cell groups. Hif1α expression was notably lower in siRNA-treated groups compared to CD133+, suggesting a reduced hypoxic response in these groups (*p* < 0.001). VHL expression also exhibited significant reductions, with particularly low levels in all siRNA-treated groups; only CD133+/Hif1α– was statistically different (*p* < 0.05). Hif2α was expressed at minimal levels across all groups, with relatively low expression in all siRNA-treated groups, indicating an overall low hypoxic response across these cell types. MIF displayed significantly decreased expression, further emphasizing that hypoxia related genes were more abundant in the CD133+ group (*p* < 0.05) (Figure 1A, Figure S2).

#### 3.1.3 Gli1 Gene Expression Altered after Gene Silencing of Hif1α, KLF4, and SHH

SHH pathway-related genes, including SHH, Gli1, SMO, and PTCH2, exhibited notable differences among cell groups. SHH expression was markedly lower in all siRNA-treated groups, highlighting a significant downregulation in SHH signaling. Gli1, a key SHH effector, was expressed at reduced levels in CD133+/Hif1α–, CD133+/KLF4–, CD133+/SHH–, and CD133+/–, indicating diminished SHH pathway activation (*p* < 0.05). SMO and specifically PTCH2 also showed statistically lower expression in CD133+/Hif1α– (*p* < 0.05 and 0.01, respectively) than CD133+, supporting the overall trend of reduced SHH pathway activity in these groups (Figure 1B, Figure S2).

#### 3.1.4 HDAC9 and EP300 Emerge as Key Molecules in the Epigenetic Marker Panel

The expression of epigenetic markers HDAC9, CREBBP, HDAC1, and EP300 varied across cell groups. HDAC9 expression was notably reduced in all siRNA-treated groups, indicating changes in epigenetic regulation (*p* < 0.05). CREBBP showed significant downregulation in only CD133+/Hif1α– (*p* < 0.01). HDAC1 expression remained consistently low across all groups, with no variation from CD133+ observed, suggesting selective changes in other epigenetic markers. EP300 was more highly expressed in CD133+ than other cell groups (*p* < 0.05). As a result, siRNA treatment may be effective on genes involved in histone acetylation, especially through HDAC9 and EP300 (Figure 1C, Figure S2).

#### 3.1.5 SMAD2 and SOX2 Have the Potential to Participate in Melanoma Stemness in the CHL-1 Cell Line

Differentiation-associated genes CD133, KLF4, NANOG, MYC, SMAD2, and MAPK1 showed significant expression alterations. CD133 expression was significantly lower in siRNA-treated groups; however, the expression reduction was not statistically different. KLF4 levels were reduced in CD133+/Hif1α– (*p* < 0.05) and CD133+/KLF4– (0.0163) (*p* < 0.001). This may be associated with the known correlation between KLF4 expression and stemness in melanoma, as previously reported in osteosarcoma.^30,31^ SMAD2 (*p* < 0.01), MAPK1 (*p* < 0.05), and SOX2 (*p* < 0.05) were significantly downregulated in CD133+/Hif1α–. These results showed that the most effective silencing group was CD133+/Hif1α–. Besides this, the most prominent findings were the reduction of gene expressions of SOX2 after siRNA treatment (*p* < 0.05) (Figure 1D, Figure S2).

#### 3.1.6 Between MMP2 and MMP9, MMP9 Was More Affected by Gene Silencing

Migration-related genes MMP2 and MMP9 display notable differences. MMP2 expression was reduced across all groups, especially in CD133+/Hif1α– (*p* < 0.05), indicating a potential decrease in migratory ability. MMP9 expression follows a similar pattern, with significant downregulation in CD133+/Hif1α– (*p* < 0.05), suggesting an overall reduction in migration potential. Reductions of gene expression were reported in both MMP2 and MMP9 in CD133+/Hif1α– and CD133+/KLF4– groups. This may be the result of MMP2 and MMP9 signaling cross talk with the Hif1α and KLF4 signals (Figure 1E, Figure S2).

#### 3.1.7 Along with FLT1 Being Influenced by Melanoma Stemness, Gene Silencing Negatively Regulates VEGFA Gene Expressions

Genes associated with vascularization, specifically, VEGFR2, FLT1, and VEGFA, were analyzed between the groups. Vascularization markers **VEGFR2**, **FLT1**, and **VEGFA** are expressed at significantly lower levels in these cell groups compared to CD133+. **VEGFR2** showed reduced expression in CD133+/Hif1α– (*p* < 0.05), indicating decreased angiogenic potential. **FLT1** expression is notably low in all of the groups. **VEGFA** was consistently low across siRNA-treated groups (*p* < 0.05) (Figure 1F, Figure S2).

### 3.2 Analysis of Protein Expression in Cellular Response to siRNA Applications

**Figure 2:**
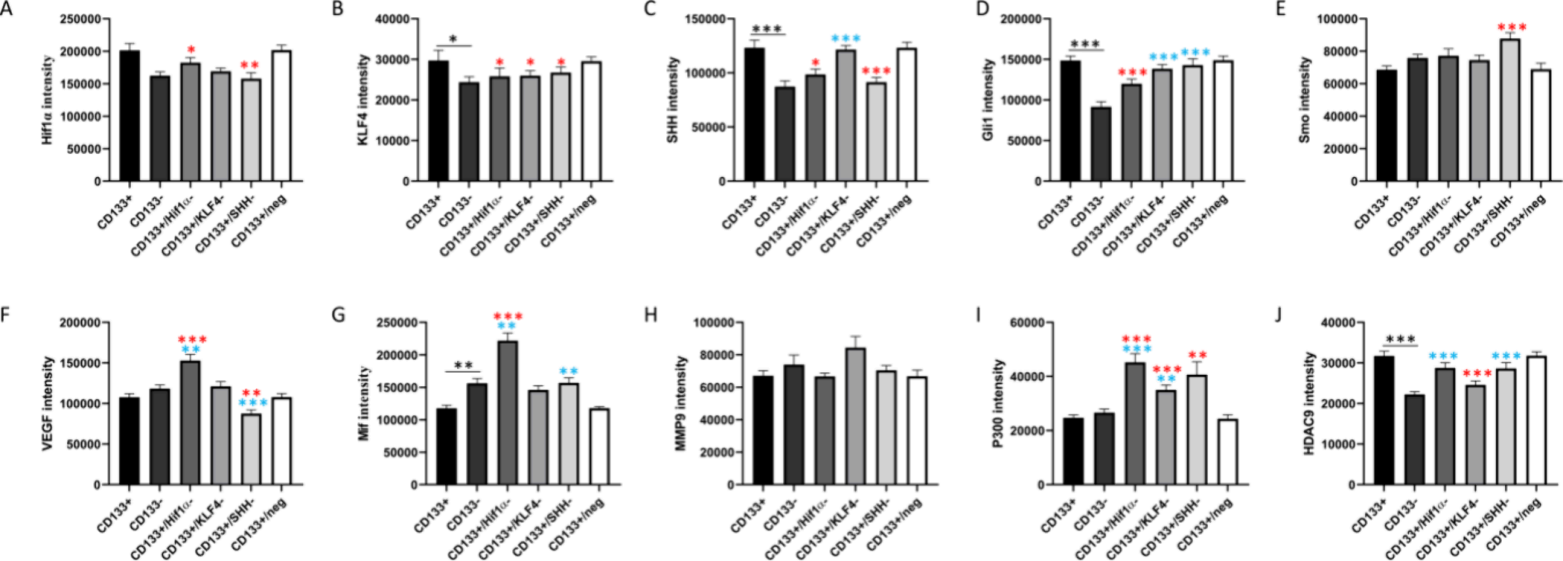
Comparison of protein expression levels following siRNA treatment targeting Hif1α, KLF4 and SHH. (A) Hif1α, (B) KLF4, (C) SHH, (D) Gli1, (E) Smo, (F) VEGF, (G) MIF, H) MMP9, (I) P300, and (J) HDAC9. Error bars represent the standard error of the mean (SEM). Statistical differences between CD133+ and CD133– cells are indicated by black stars. Red stars indicate statistical differences between CD133+ and siRNA-treated groups, while blue stars represent differences between CD133– and siRNA-treated groups. Statistically significant difference was defined as *p < 0.05, **p < 0.01, and ***p < 0.001.

Protein intensity values are graphically presented in Figure 2 corresponding to Hif1α siRNA, KLF4 siRNA, and SHH siRNA treatments. Immunofluorescent images of the cells are provided in the Supporting Information (Figure S3–S12).

#### 3.2.1 Silencing SHH Reduces Hif1α Expression in CD133+ Melanoma Cells

CD133+ cells without any silencing showed high Hif1α intensity, suggesting that CD133+ cells naturally have elevated Hif1α expression. Silencing KLF4 in CD133+ cells resulted in higher HIF1α intensity than both the CD133+/SHH– and CD133– groups, indicating that KLF4 silencing has a less suppressive effect on Hif1α expression compared to SHH silencing. Silencing SHH in CD133+ cells appear to significantly reduce Hif1α expression compared to other groups (*p* < 0.01) (Figure 2, Figure S3).

#### 3.2.2 Silencing SHH and Hif1α Tends to Reduce KLF4 Expression in CD133+ Melanoma Cells

CD133+ cells exhibited the highest KLF4 expression, highlighting the strong association between CD133 character and KLF4 levels, with statistical difference between CD133+ and CD133– cell groups (*p* < 0.05). Silencing KLF4 in CD133+ cells decreased KLF4 protein intensity, confirming the effectiveness of the silencing (*p* < 0.05). Silencing SHH and Hif1α in CD133+ cells also reduced KLF4 intensity, with Hif1α silencing having a more pronounced effect than SHH. CD133– cells had the lowest KLF4 expression, suggesting the importance of the CSC character in maintaining high KLF4 levels (Figure 2, Figure S4).

#### 3.2.3 Silencing KLF4 and Hif1α Impacts SHH Expression in CD133+ Melanoma Cells, While CD133+ Cells Naturally Exhibit Higher SHH Intensity Compared to CD133– Cells

CD133+ cells exhibited the highest SHH expression, indicating a strong association between CD133 and SHH levels. CD133– cells had the lowest SHH intensity, highlighting the CSC character in maintaining high SHH expression (*p* < 0.001). Silencing KLF4 in CD133+ cells resulted in high SHH intensity, and the protein level was comparable to CD133+. Silencing Hif1α in CD133+ cells moderately reduced SHH intensity (*p* < 0.05), implying Hif1α’s role in positively regulating SHH expression. Silencing SHH in CD133+ cells showed a decrease in SHH intensity compared to CD133+ cells (*p* < 0.001) (Figure 2, Figure S5).

#### 3.2.4 Gli1 Protein Expression Was Higher in CD133+ Cells Compared to CD133– Cells Even following siRNA Applications

CD133+ cells exhibited the highest Gli1 expression, highlighting the association between CD133 and elevated Gli1 levels. CD133– cells showed the lowest Gli1 intensity, indicating minimal Gli1 expression (*p* < 0.001). Silencing Hif1α in CD133+ cells decreased Gli1 intensity, suggesting roles for Hif1α in positively regulating Gli1 expression (Figure 2, Figure S6).

#### 3.2.5 Smo Protein Expression Tended to Increase after siRNA Applications, Especially after SHH siRNA Treatments

Following siRNA treatment, an increase in Smo protein expression was observed, with the CD133+/SHH– group exhibiting significantly different Smo protein levels (*p* < 0.001) (Figure 2, Figure S7).

#### 3.2.6 VEGF Protein Expression Responded Differently after siRNA Treatment

There was no significant difference in VEGF protein expression between the CD133+ and CD133– groups. However, both CD133+ and CD133– groups exhibited higher VEGF expression levels compared to the CD133+/SHH– group (*p* < 0.01 and 0.001, respectively). Silencing Hif1α in CD133+ cells (CD133+/Hif1α−) resulted in a significant increase in VEGF protein expression relative to the CD133+ (*p* < 0.001) and CD133– (*p* < 0.01) groups. Additionally, silencing KLF4 in CD133+ cells led to a moderate increase in VEGF intensity. VEGF protein expression exhibited differential responses following siRNA treatments; specifically, silencing SHH in CD133+ cells resulted in decreased VEGF expression when compared to both the CD133+ and CD133– groups (*p* < 0.01 and 0.001, respectively) (Figure 2, Figure S8).

#### 3.2.7 MIF Protein Expression Increased after Hif1α and SHH siRNA Application

MIF protein levels were significantly higher in the CD133– group compared to the CD133+ group (*p* < 0.01). The silencing of Hif1α in CD133+ cells led to a notable increase in MIF intensity, emphasizing a strong regulatory influence of Hif1α on MIF expression (*p* < 0.001 for CD133+/Hif1α– versus CD133+ and *p* < 0.01 for CD133+/Hif1α– versus CD133+/neg). Additionally, MIF protein expression was elevated following SHH siRNA application compared to the CD133– group (*p* < 0.01) (Figure 2, Figure S9).

#### 3.2.8 KLF4, SHH, and Hif1α Silencing Does Not Directly Affect MMP9 Expression in 24 h

The expression levels of MMP9 protein were similar between the CD133+ and CD133– groups as well as the siRNA-treated groups. However, there was a slight increase in MMP9 expression following KLF4 silencing (Figure 2, Figure S10).

#### 3.2.9 P300 Protein Expression Increased after All Three siRNA Applications

The CD133– group showed moderate P300 intensity comparable to that of CD133+ cells. Silencing KLF4, SHH, and Hif1α, as well as other siRNA treatments, led to increased P300 protein expression with significant differences observed between the CD133+ and CD133– groups. P300 protein expression was elevated following all three siRNA applications (Figure 2, Figure S11).

#### 3.2.10 HDAC9 Protein Expression Tends to Decrease after siRNA Applications

HDAC9 protein levels were significantly higher in the CD133+ group compared to the CD133– cell group (*p* < 0.001). The CD133+/KLF4– group showed HDAC9 levels comparable to the CD133– group but was statistically distinct from both the CD133+/Hif1α and CD133+/SHH– groups (*p* < 0.001). HDAC9 protein expression tended to decrease following siRNA applications (Figure 2, Figure S12).

#### 3.2.11 Gli1, HDAC9, and MMP9 Secretion Did Not Differ after Silencing Hif1α, KLF4, and SHH

To determine whether the cell groups released the relevant proteins from the cell, an ELISA was conducted.

**Figure 3:**
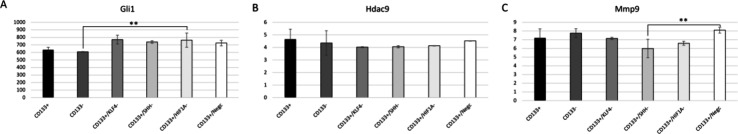
ELISA measurement of protein secretion in CD133+ and CD133– cells following gene silencing. (A) Gli1, (B) HDAC9, and (C) MMP9 protein secretion was quantified in cell groups after siRNA treatment targeting Hif1α, KLF4, and SHH. Gli1 secretion was significantly higher in the CD133+/Hif1α– groups compared to CD133– cells (*p* < 0.01). (B) HDAC9 secretion was highest in the CD133+ group, with no significant difference observed between CD133+ and CD133– cells. Following siRNA treatments, HDAC9 secretion levels remained similar across the experimental groups. (C) MMP9 secretion was highest in the CD133+/– cells, with a statistically significant difference compared to the CD133+/SHH– group (*p* < 0.01). Data are presented as mean ± SEM, with statistical significance evaluated using a one-way ANOVA with post hoc Tukey’s test. Asterisks indicate *p* < 0.05 and *p* < 0.01.

Gli1 secretion was highest in the CD133+/KLF4– (770.54 pg/mL) and CD133+/Hif1α– cell groups (763.403 pg/mL). A statistical difference was observed between CD133+/Hif1α– and CD133– (607.035 pg/mL) (*p* < 0.01). Following CD133+/KLF4– and CD133+/Hif1α–, CD133+/– showed the highest secretion (724.645 pg/mL). KLF4, SHH, and Hif1α siRNA treatment increased Gli1 secretion (Figure 3A). HDAC9 secretion was the highest in the CD133+ group. It was demonstrated that CD133+ and CD133– cells were not distinct from one another (4.65 and 4.355 pg/mL). After siRNA applications, the level of HDAC9 secretion was similar (Figure 3B). The maximum MMP secretion was seen in the CD133+/– cells (8.09 pg/mL). Between the CD133+/SHH– group and the CD133+/– group, there was a statistically significant difference (*p* < 0.01). After siRNA treatment, MMP9 secretion was consistent (Figure 3C). GLI1, HDAC9, and MMP9 secretion did not statistically differ after silencing Hif1α, KLF4, and SHH.

### 3.3 Cell Cycle Analysis Revealed the Critical Roles of Hif1α and KLF4 in the G0/G1 and S Phases, While SHH Was Found to Be Crucial in the G2/M Phase of Melanoma CSCs

**Figure 4:**
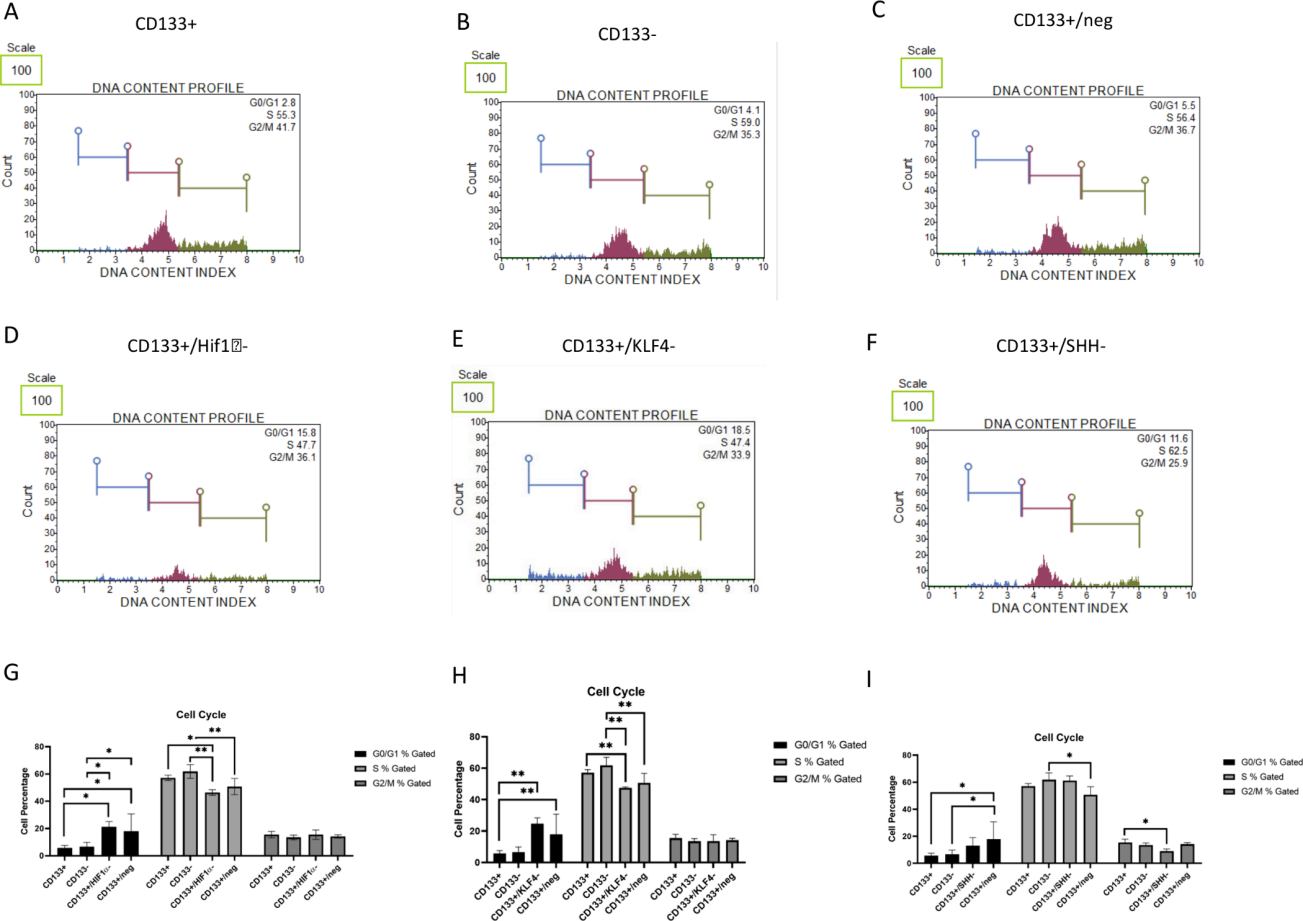
Cell cycle stage distribution in CD133+ and CD133– cells following siRNA treatments is compared among cell groups. Bench-top flow cytometry analysis of cell cycle distribution in CD133+, CD133–, and siRNA-treated cells. The percentage of cells in the G0/G1, S, and G2/M phases is shown. The sample percentage distributions are shown as (A) CD133+, (B) CD133–, (C) CD133/–, (D) CD133/Hif1α–, (E) CD133/KLF4–, and (F) CD133/SHH–. The cell cycle phase comparison graphs for (G) Hif1α, (H) KLF4, and (I) SHH siRNA-treated groups are provided. G0/G1 phase distribution was significantly higher in the CD133+/Hif1α– and CD133+/KLF4– groups compared to CD133+ and CD133– cells (*p* < 0.05 and 0.01). S phase distribution showed a significant decrease in CD133+/Hif1α– cells (*p* < 0.01) and CD133+/KLF4–. No significant difference was observed in the G2/M phase except for CD133+/SHH– cells, which exhibited a decrease (*p* < 0.05). Error bars represent the standard error of the mean. Statistical analysis was performed using one-way ANOVA, and *p* < 0.05 was considered statistically significant.

In this study, we evaluated the cell cycle by analyzing the distribution of cells across the G0/G1, S, and G2/M phases (Figure 4A–F). In the G0/G1 phase, there was no difference between CD133+ and CD133– cells (2.9 and 3.4%); however, silencing Hif1α (CD133+/Hif1α−) resulted in a significant increase (16.2%; *p* < 0.05), and the CD133+/Hif1α– cell group was statistically different from CD133+ and CD133– cell groups (*p* < 0.05) (Figure 4G). Similarly, KLF4 silencing led to an increase in the G0/G1 phase (18.6%) (*p* < 0.01) (Figure 4H), while SHH silencing resulted in a percentage increase of 10.4% (Figure 4I). The negative siRNA-treated group (CD133+/–, 7.03%) was statistically different from CD133+ and CD133– cell groups (*p* < 0.05). Comparatively, in the S phase, there was no difference between CD133+ and CD133– cells (54.3%, 60.1%), while CD133+/Hif1α– cells exhibited a significant decrease (48.2%) compared to CD133– cells (*p* < 0.01) (Figure 4). Furthermore, while CD133+/KLF4 cells showed an increase (61.3%) compared to CD133+, CD133+/SHH (49.4%) cells remained similar to the CD133+ cell group. Conversely, no differences were observed between cell groups in the G2/M phase with Hif1α and KLF4 silencing (Figure 4G,H), whereas SHH silencing led to a decrease (*p* < 0.05) (Figure 4I).

## 4 Discussion

Melanoma, a highly aggressive type of skin cancer, presents significant treatment challenges due to its low survival rate and resistance to multiple drugs.^36,37^ Within the tumor cell hierarchy, CSCs are a distinct population of undifferentiated cells with heightened tumorigenicity, metastatic ability, self-renewal capabilities, and therapy resistance.^38^ Here, we aim to identify differences between CSCs and NCSCs in the presence of the extracellular matrix and determine if manipulating genes associated with hypoxia and differentiation alters the characteristics of CSCs. In this study, the CD133 marker was considered to represent the CSC. The cells obtained by flow cytometry maintained the stem cell characteristics on the Matrigel over the CD133 marker at the gene expression level (Figure 1D). Additionally, the stemness feature (CD133 level) decreased after siRNA treatment (Figure 1D). This outcome indicates that specific signaling pathways have the potential to reduce CSC character, highlighting the potential of transitioning cells into NCSCs through alterations in stem cell characteristics.

CSCs’ characteristics have been manipulated using various extracellular matrices to mimic their *in vivo* environment. Bonturi et al. investigated laminin, fibronectin, vitronectin, and other intercellular agents separately in culture media and evaluated their protease efficiency.^39^ Matrigel, particularly in studies involving the melanoma CHL-1 cell line, has shown significant effects on cell invasion potential both on culture surfaces and in invasion studies. These findings are commonly used to explore the cellular differentiation potential.^39−41^ Although this study mentions the interaction between CSCs and the extracellular matrix (here, Matrigel), it is important to clarify that the primary focus of our experiments was on the modulation of CSC characteristics via gene silencing (Hif1α, KLF4, and SHH). The primary objective of our study is to examine alterations in cancer-related signaling molecules by silencing Hif1α, KLF4, and SHH genes.

Hif1α is well-known to be regulated by hypoxic conditions, where it activates various pathways that promote tumor survival and progression. Under hypoxic conditions, Hif1α induces the expression of genes involved in angiogenesis, metabolism, and cell survival, which are essential for the adaptation of cancer cells to low oxygen levels. However, although Hif1α is predominantly regulated by hypoxia, recent studies have shown that Hif1α also plays significant roles under normoxic conditions. In our study, we hypothesized that even in the absence of hypoxia, Hif1α’s basal expression may influence the maintenance of cancer stem cells (CSCs). Basal levels of Hif1α have been shown to affect various cellular processes, such as stemness, proliferation, and drug resistance, in melanoma and other cancer types. These findings suggest that Hif1α’s function is dependent not solely on hypoxia but also on its baseline activity in the cell, influencing key pathways that regulate CSC properties.^42−45^

CSCs are more prominent in tumor masses, particularly in cell lines and spheroid models. Based on the hypothesis of maintaining stem cell capacity, the Hif1α gene of CSCs has been silenced in previous studies.^46,47^ Here, in the hypoxia panel, the most prominent finding revealed that NCSCs showed a decreased expression of the Hif1α gene and, upon gene silencing of KLF4 and SHH, affected the Hif1α gene expression (Figure 1, Figure S2). Besides, silencing Hif1α gene expression decreases the VHL gene expression. This aligns with Hif1α and VHL relation, wherein VHL stabilizes Hif1α under normoxic conditions. Similar patterns were observed in VHL-deficient renal cancer cells, suggesting the impact on the downstream signaling of Hif1α.^48^ The Hif1α protein interacts with the CD133 gene promoter, increasing the frequency of CD133+ glioma, colon, and pancreatic cell CSCs via OCT4 and SOX2.^49−54^ Additionally, a cytoplasmic correlation between Hif1α and CD133 was observed,^55^ where CD133 can influence HIF1α expression and facilitate its nuclear translocation during hypoxia.^56^ A previous research has demonstrated a correlation between NANOG and OCT4 expression and Hif1α levels in prostate cancer cells.^57^ Although Hif1α and NANOG showed similar trends in the prostate cancer study, decreased Hif1α gene expression did not significantly change NANOG gene expression in melanoma CSC.

SHH plays a crucial role in cell differentiation and tissue polarity during embryonic development with mutations in SHH pathway genes observed in melanoma patients.^58^ Our study highlighted Gli1 as a potential target, particularly evident with KLF4 silencing, leading to a significant decrease in Gli1 expression. This indicates that there may be Gli1 and KLF4 interactions specific to melanoma or melanoma stem cells. The focus on Gli1 was based on its established role as a key downstream effector of SHH signaling in melanoma, particularly in the regulation of stemness and tumor progression. Although Gli1 was the primary gene analyzed in our study, further investigation into additional SHH-related genes would be valuable to provide a more comprehensive understanding of SHH pathway dynamics in stem cell biology. Furthermore, PTCH2 expression was significantly affected by gene silencing, particularly with Hif1α silencing, suggesting a potential interaction of Hif1α and PTCH2. Protein expression analysis revealed higher SHH and Gli1 levels in CD133+ cells compared to those in CD133– cells, while Smo expression was comparable.

In our study, Hif1α silencing correlated with reduced expression of EP300, involved in histone acetylation, and HDAC9, implicated in angiogenesis and cancer.^59−62^ P300 protein levels increased with all three siRNA applications, while HDAC9 tended to decrease. Furthermore, all three siRNA treatments resulted in lower levels of HDAC9 protein secretion compared with the CD133+ cell group. Specifically, the KLF4 siRNA application showed the lowest amount of secreted HDAC9.

MMP2 and MMP9 are key enzymes involved in breaking down the extracellular matrix under physiological conditions, and studies have identified them as a potential markers for breast^63^ and melanoma^64,65^ cancers. In our study, when comparing CSCs and NCSCs from the CHL-1 cell line, we observed lower MMP expression in the NCSC, with siRNA treatments leading to a decrease in MMP2 and MMP9 gene expression. This reduction aligns with the disruption in KLF4, SHH, and Hif1α genes, indicating their efficacy against cancer cells. Previous studies have shown a decrease in MMP proteins after SHH siRNA treatment in gastric and liver cancer cells.^66,67^ Similarly, in glioblastoma research, Hif1α, MMP9, and VEGF proteins displayed similar trends, potentially explaining the decrease in VEGF and MMP9 gene expression with Hif1α silencing.^68^ Interestingly, our protein-level analysis revealed a similar protein expression pattern after 24 h of incubation with siRNA.

As a hallmark of cancers, a cancer-specific network of blood vessels is required for melanoma to survive and grow.^69^ Research on melanoma cell lines has highlighted the significance of VEGFR2 and VEGFA in cell metastasis, with VEGFR2 playing a dominant role in invasion due to its higher expression level.^70^ In melanoma cells, autocrine or paracrine VEGFR-1 (FLT1) activation increases cancer cell survival, cell migration, invasion, and chemotherapy resistance. In a study performed in A375 and M14 melanoma cell lines, it has been shown to reduce invasion capacity following with VEGFR-1 inhibition.^71^ VEGFR1 gene expression decreased after Hif1α, KLLF4, and SHH silencing. VEGF protein intensity showed a decrease with SHH silencing correlated with the transcriptional level, which indicated direct relation with VEGF and SHH in CD133+ melanoma cells.

Cell cycle regulation is one of the important hallmarks of tumor resistance. Several studies showed that cell cycle arrest was operated via Hif1α.^72−75^ Our study observed the G0/G1 phase arrest after Hif1α silencing, consistent with the literature. Additionally, Hif1α and KLF4 siRNA treatment reduces the S phase compared to the CSCs and NCSC, indicating Hif1α’s specific targeting potential. SHH silencing impacted the G2/M phase, aligning with the literature on SHH signaling.^76^ Further studies are required for exploring vascularization, glycolytic pathways, and mitochondrial processes in the tumor microenvironment.

## 5 Conclusions

This study provides valuable insights into the biology of melanoma CSCs by investigating the effects of silencing three key genes—Hif1α, KLF4, and SHH—on their stem-like characteristics with the presence of an extracellular matrix. Our findings reveal that silencing these genes leads to significant reductions in stemness-related features, including hypoxia responsiveness, migratory potential, and epigenetic regulation. Notably, Hif1α silencing was associated with a marked reduction in hypoxia-related gene expression, while SHH silencing significantly decreased the expression of Gli1, a downstream effector of SHH signaling, positioning it as a promising therapeutic target. While our study primarily focused on Gli1 as a key effector in the SHH pathway, we recognize that future research should include a broader analysis of SHH-related genes. This will provide a more holistic view of the SHH pathway and its regulation of melanoma CSCs. Additionally, changes in epigenetic markers such as HDAC9 and EP300 underscore their crucial role in maintaining stemness and regulation of gene expression in melanoma CSCs. Importantly, these siRNA-based interventions reprogrammed CSCs into a phenotype distinct from both traditional CSCs and NCSCs, revealing the plasticity of these cells and their potential for therapeutic targeting. Our results emphasize the critical importance of targeting key signaling pathways, including hypoxia and SHH, in melanoma CSCs. Furthermore, the study highlights the value of mimicking the tumor microenvironment in experimental models to uncover more accurate therapeutic insights. By demonstrating the potential for siRNA to modulate pathways linked to tumor progression and stem cell behavior, this work offers promising directions for the development of novel melanoma therapies.

## Supporting Information Available

Additional data supporting the findings of this study, including flow cytometry plots, spheroid formation images, AFM/SEM images of Matrigel-coated surfaces, heatmaps of gene expression profiles, and representative immunofluorescence images of key proteins (DOCX)

## Notes

Not applicable. There are no humans or animals directly involved in this study.
