# Supplementary material for: Modulating Cancer Stem Cell Characteristics in CD133+ Melanoma Cells through Hif1α, KLF4, and SHH Silencing
Source: ACS Omega. 2025 Apr 18;10(16):16804–14. doi: 10.1021/acsomega.5c00799 (PMC12044452; doi:10.1021/acsomega.5c00799)
Supplement: Supplementary file 1 — ao5c00799_si_001.docx [file ao5c00799_si_001.docx]

**Modulating cancer stem cell characteristics in CD133+ melanoma cells through *Hif1α*, KLF4, and SHH silencing**

Berrin Ozdil^1,2,3^, Cigir Biray Avci^4^, Duygu Calik-Kocaturk^5^, Volkan Gorgulu^2^, Aysegul Uysal^2^, Gunnur Guler^3^, Nefise Ülkü Karabay Yavaşoğlu^6^, Huseyin Aktug^2*^

^1^Department of Histology and Embryology, Faculty of Medicine, Suleyman Demirel University, 32260, Isparta, Turkey

^2^ Department of Histology and Embryology, Faculty of Medicine, Ege University, 35100, Izmir, Turkey

^3^ Department of Physics, Biophysics Laboratory, Izmir Institute of Technology, 35430, Izmir, Turkey

^4^ Department of Medical Biology, Faculty of Medicine, Ege University, 35100, Izmir, Turkey

^5^ Dr. İsmail Fehmi Cumalioglu City Hospital, 59030, Tekirdağ, Turkey

^6^ Department of Biology, Faculty of Science, Ege University, 35100, Izmir, Turkey

^*^Corresponding author: Huseyin Aktug: [huseyin.aktug@ege.edu.tr](mailto:huseyin.aktug@ege.edu.tr)

**Supplementary Figures**

**
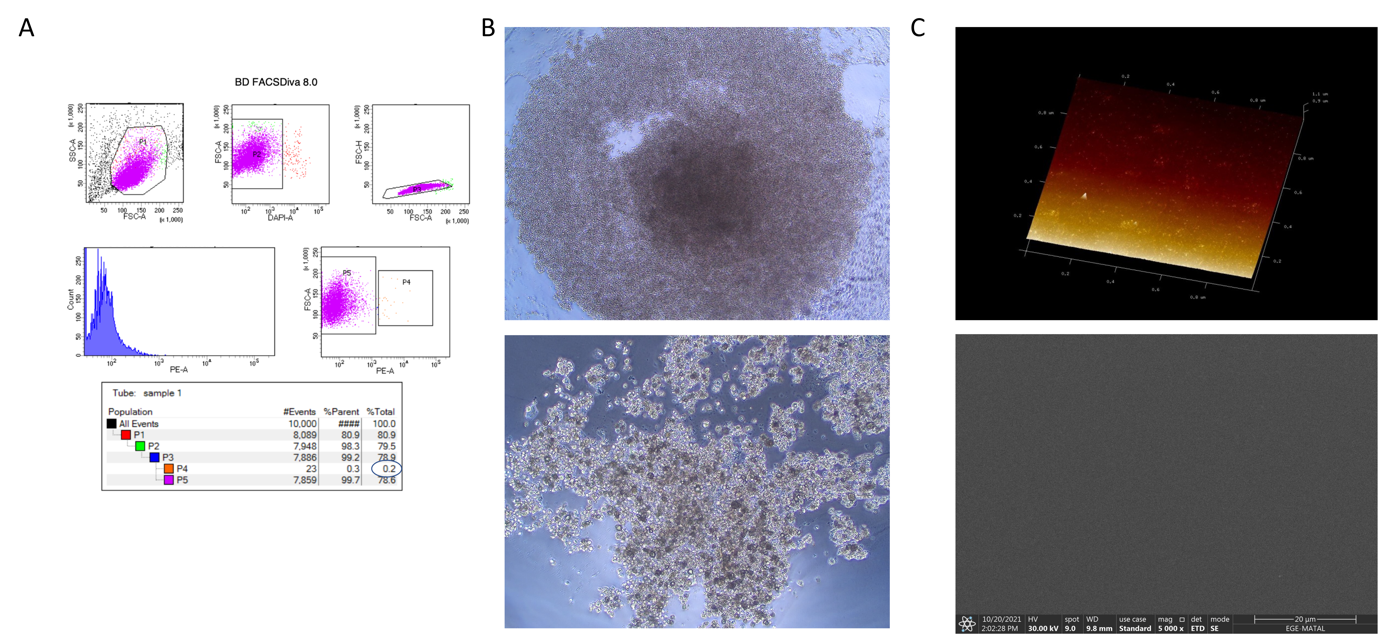
**

**Figure S1:** A) Flow cytometry analysis of the CHL-1 cell line. B) Sphere formation assay following FACS analysis. The upper image represents the CD133+ cell group, while the lower image shows the CD133− cell group (4x magnification). C) Morphological characterization of the Matrigel surface using AFM and SEM techniques.

**
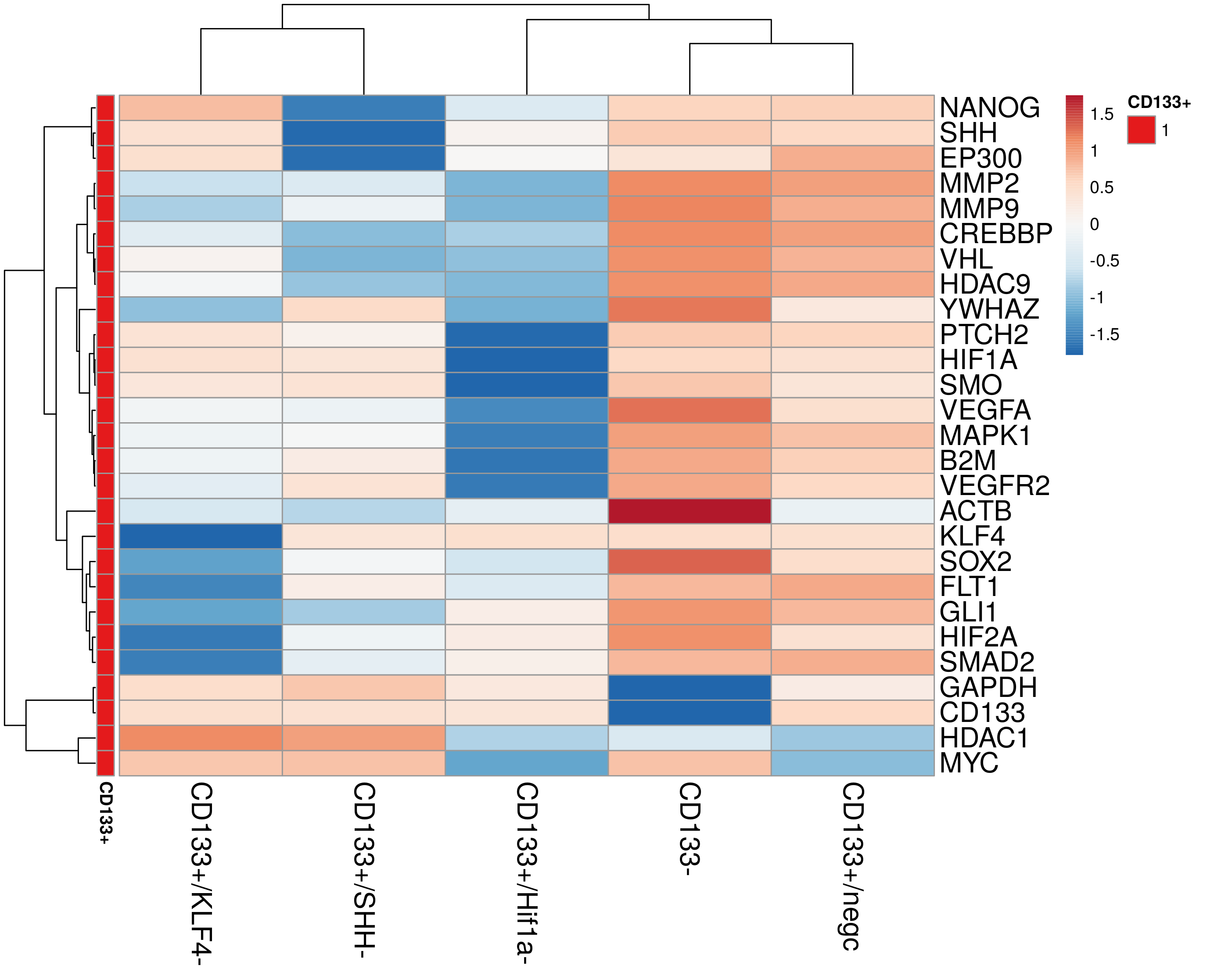
**

**Figure S2:** Heatmap illustrating gene expression levels across different cell groups categorized by CD133 status and associated molecular markers according to their fold regulations (Fold regulation= -1/Fold change). Values represent log2 fold changes relative to CD133+ group and housekeeping genes (ACTB, GAPDH, B2M, YWHAZ). Highlighted pathways and markers include hypoxia (Hif1α, VHL, Hif2α), SHH pathway (SHH, GLI1, SMO, PTCH2), epigenetic markers (HDAC9, CREBBP, HDAC1, EP300), differentiation factors (CD133, KLF4, NANOG, MYC, SMAD2, MAPK1, SOX2), migration (MMP2, MMP9), and vascularization (VEGFR2, FLT1, VEGFA).

**
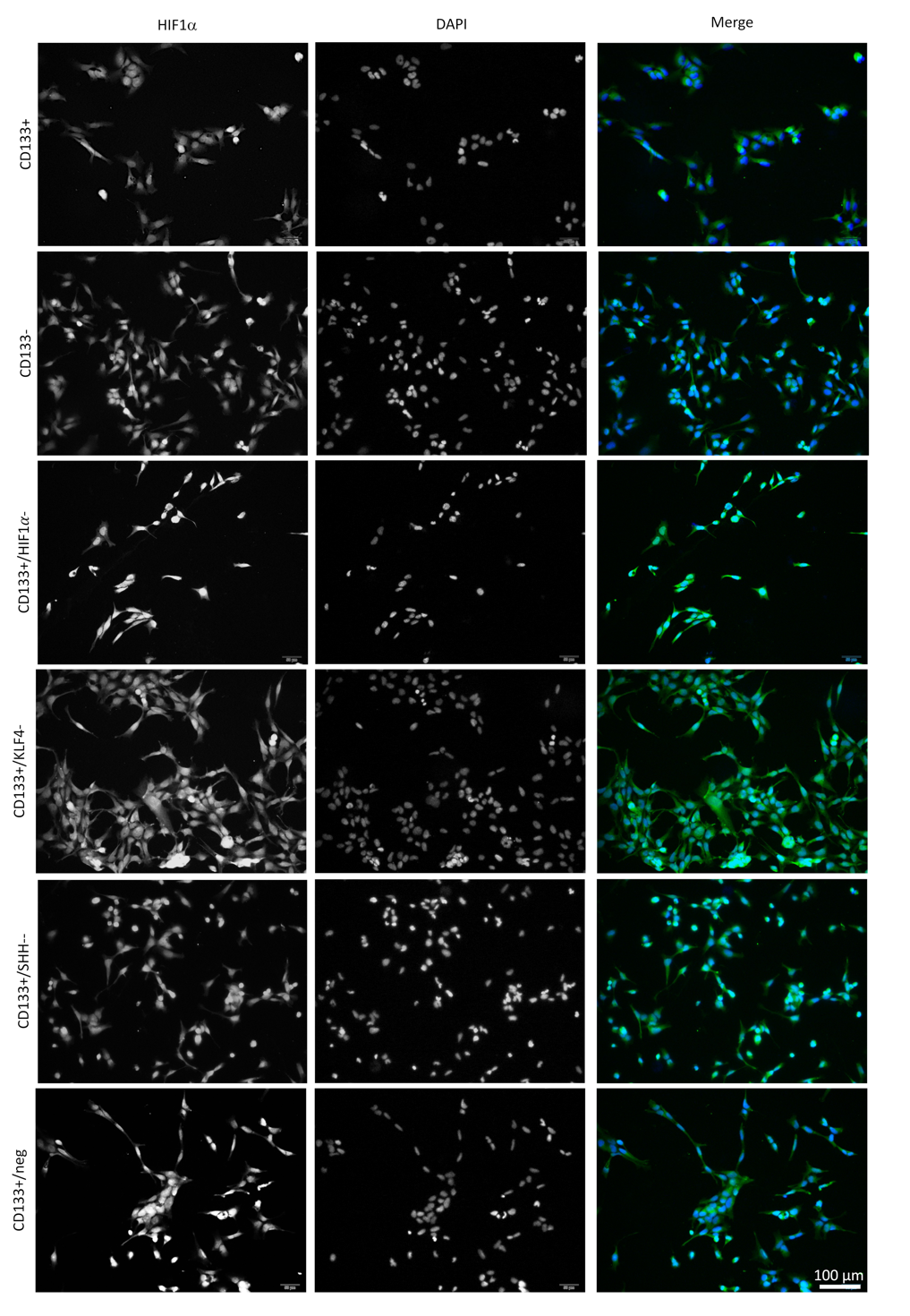
**

**Figure S3:** The representative images of Hif1α protein staining in grayscale, with DAPI staining indicating nuclei. In merged images, Hif1α is green and nuclei in blue. Following SHH silencing, there is a notable reduction in Hif1α protein expression.

**
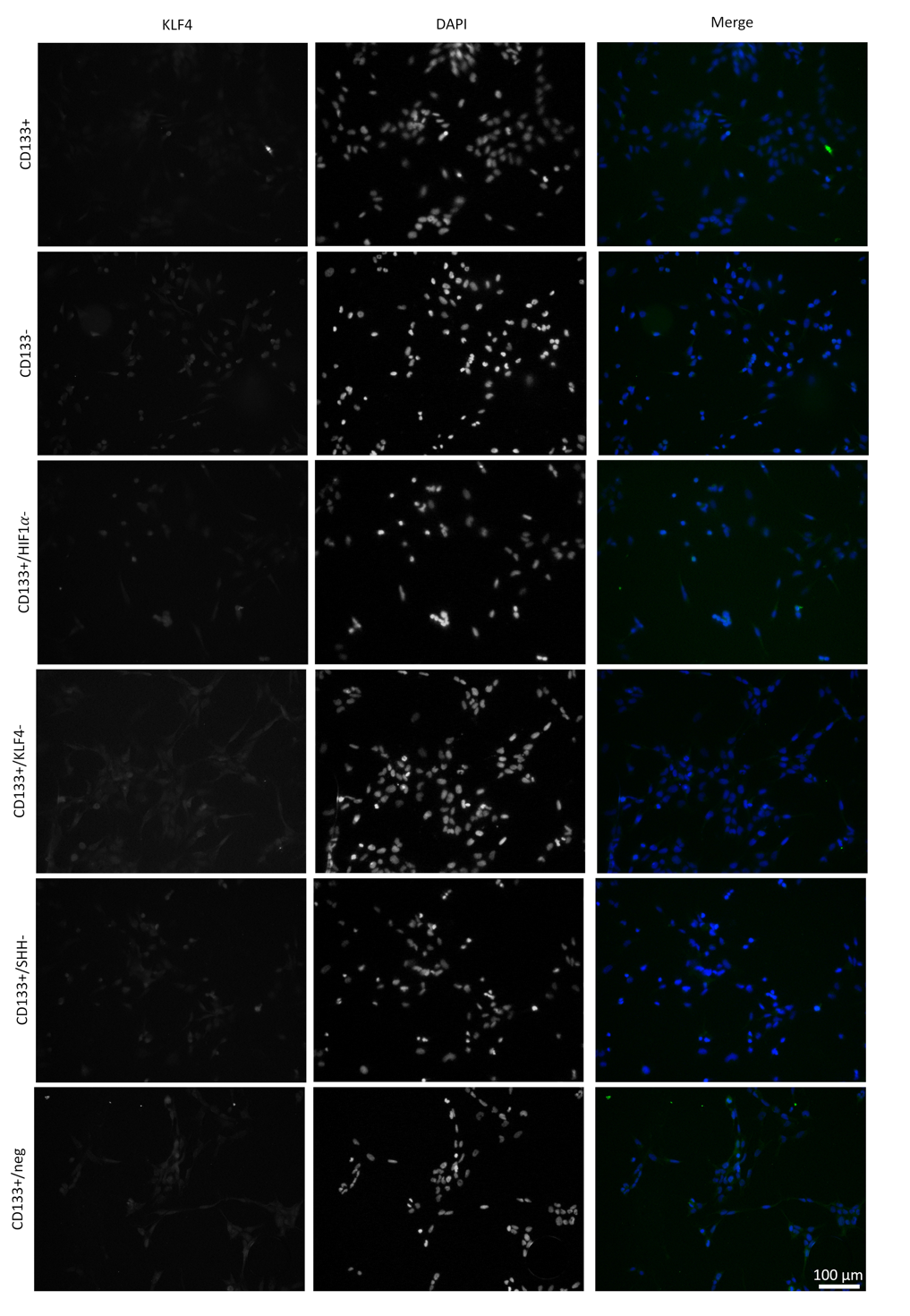
**

**Figure S4:** Representative images of KLF4 protein staining in grayscale, with DAPI staining indicating nuclei. In merged images, KLF4 is green and nuclei are blue. All experimental groups exhibit similar KLF4 protein intensity.

**
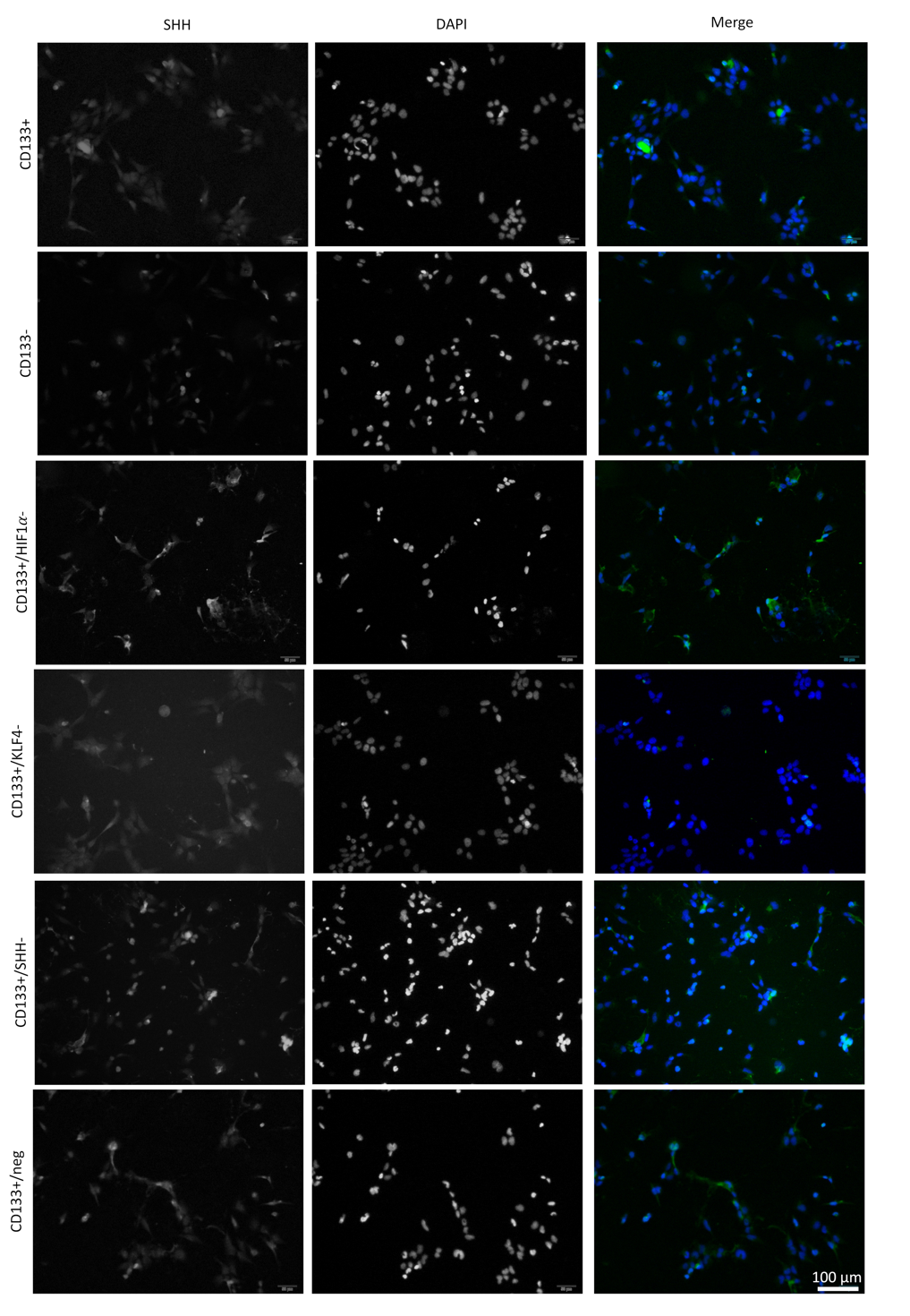
**

**Figure S5:** Representative images of SHH protein staining in grayscale, with DAPI staining indicating nuclei. In merged images, SHH is green and nuclei are blue. The CD133+ group exhibits elevated SHH expression compared to CD133- cell group. After silencing both HIF1α and SHH, SHH expressions decrease.

**
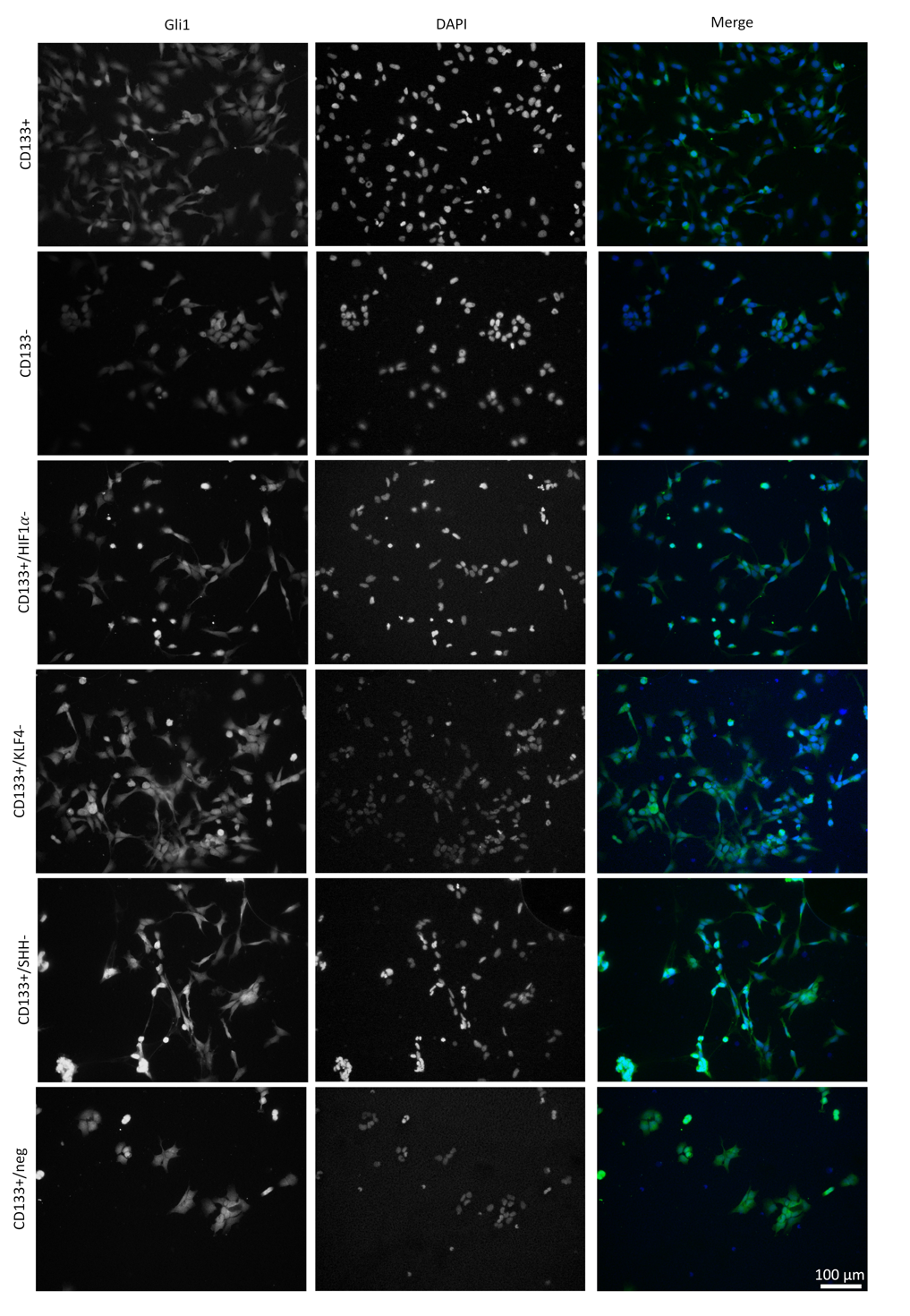
**

**Figure S6:** Representative images of Gli1 protein staining in grayscale, with DAPI staining indicating nuclei. In merged images, Gli1 is green and nuclei are blue. The CD133+ group exhibits higher Gli1 expression than CD133- cell group. After HIF1α siRNA treatment, Gli1 expression decreased.

**
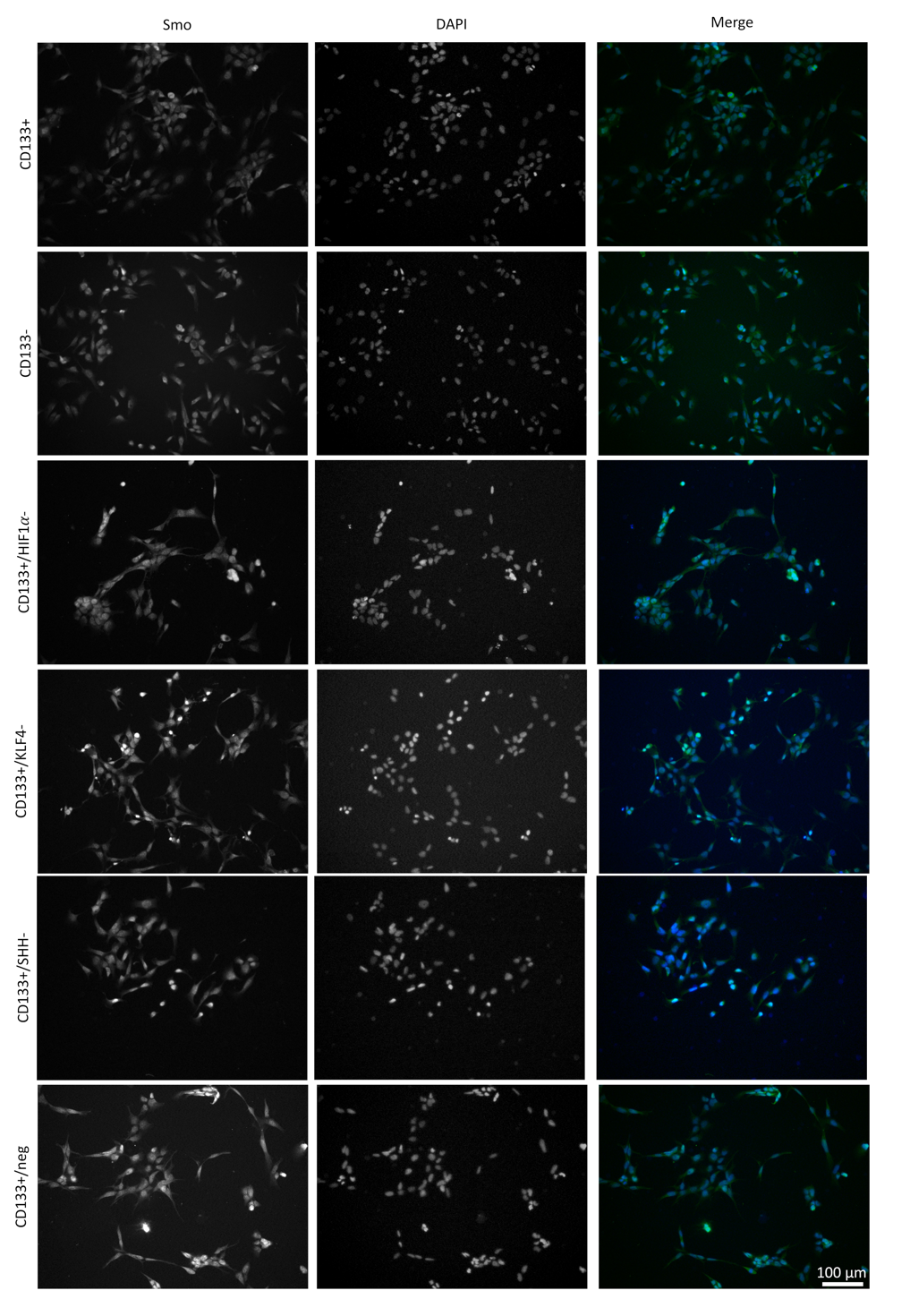
**

**Figure S7:** Representative images of Smo protein staining in grayscale, with DAPI staining indicating nuclei. In merged images, Smo is green and nuclei are blue. After SHH siRNA treatment, Smo expression increases.

**
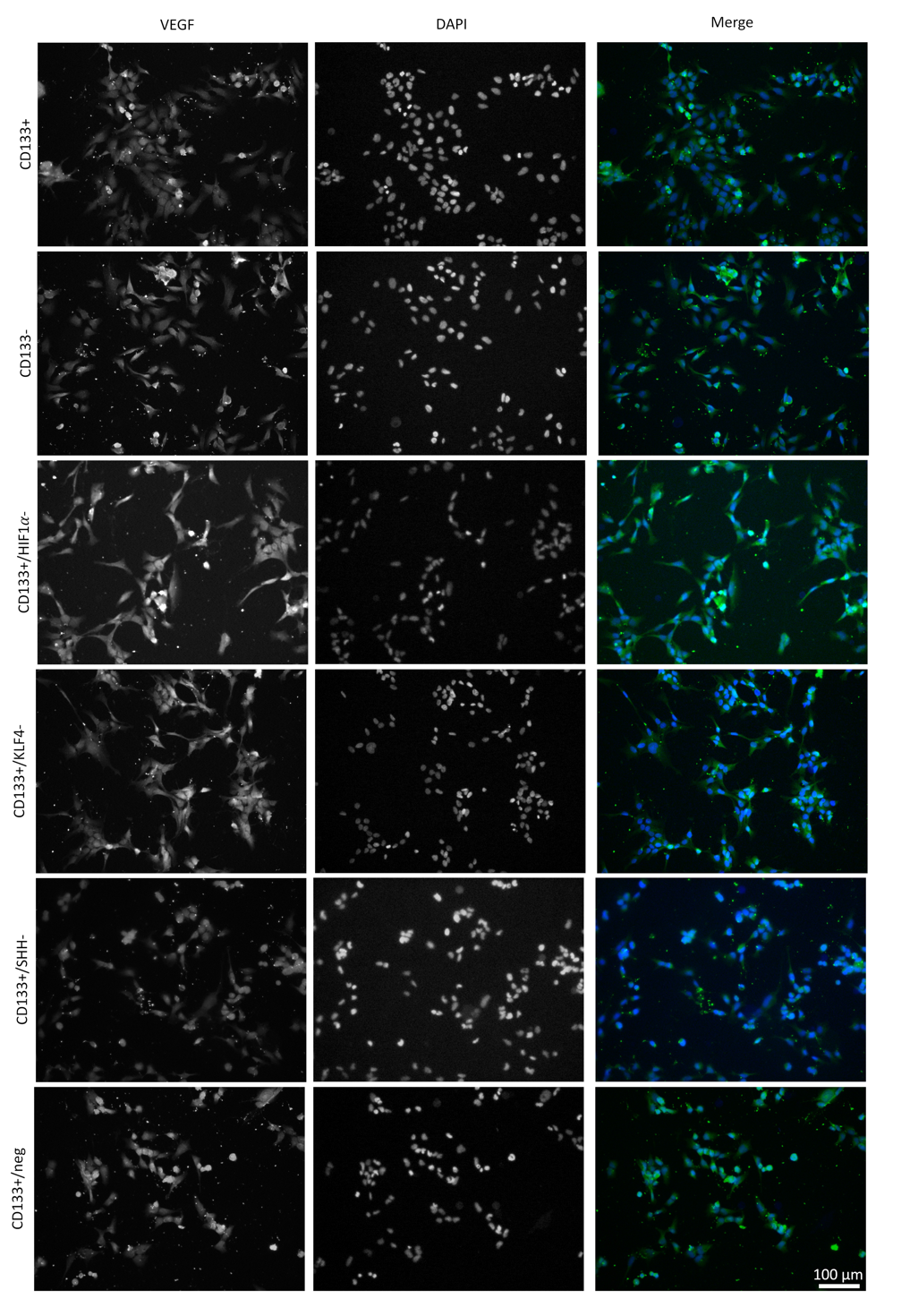
**

**Figure S8:** Representative images of VEGF protein staining in grayscale, with DAPI staining indicating nuclei. In merged images, VEGF is green and nuclei are blue. After HIF1α siRNA treatment, Gli1 expression decreases.

**
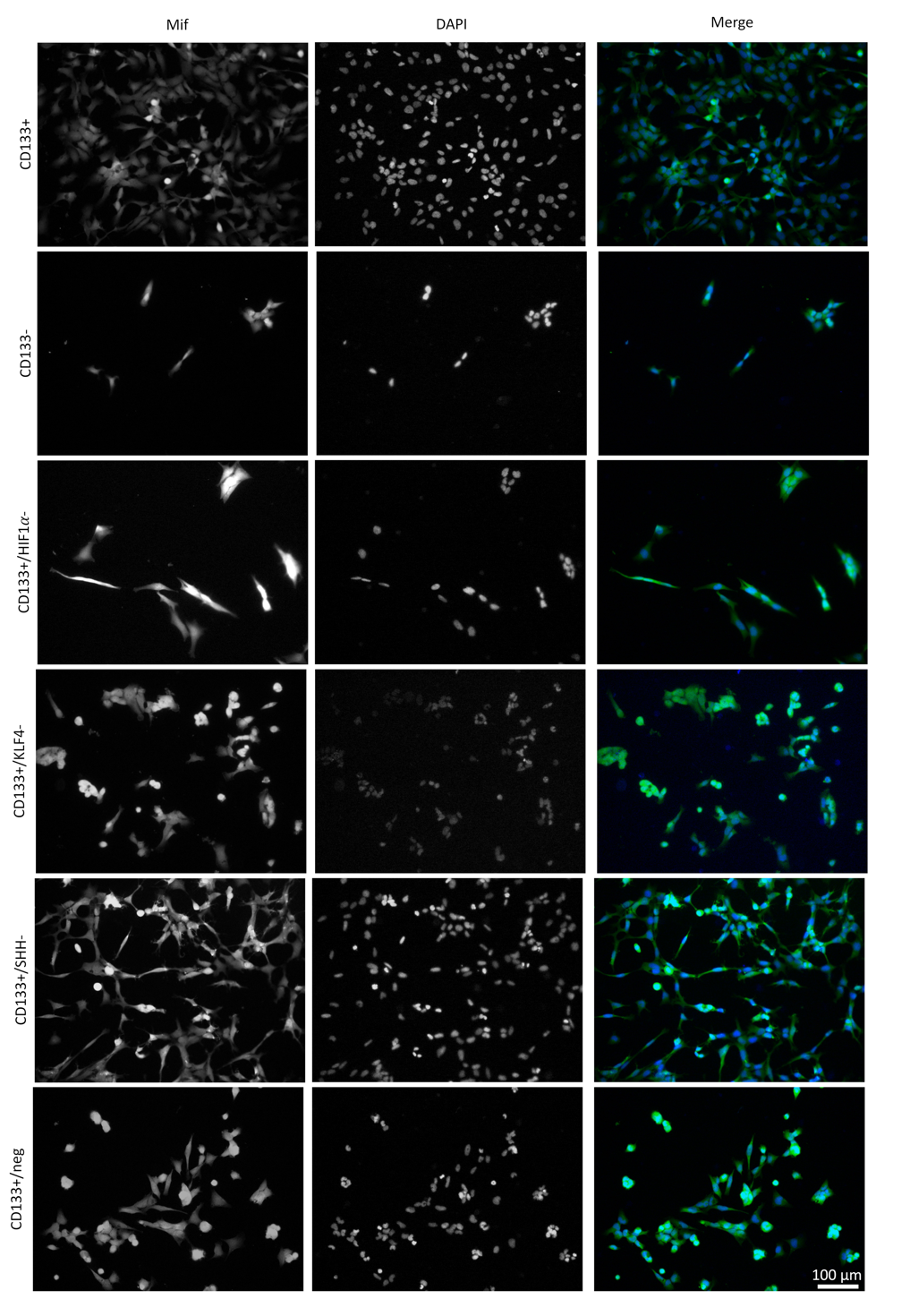
**

**Figure S9:** Representative images of Mif protein staining in grayscale, with DAPI staining indicating nuclei. In merged images, Mif is green and nuclei are blue. The CD133- group exhibits elevated Mif expression compared to CD133+ cell group. After HIF1α siRNA treatment, Mif expression increases, while SHH siRNA decreases Mif expression.

**
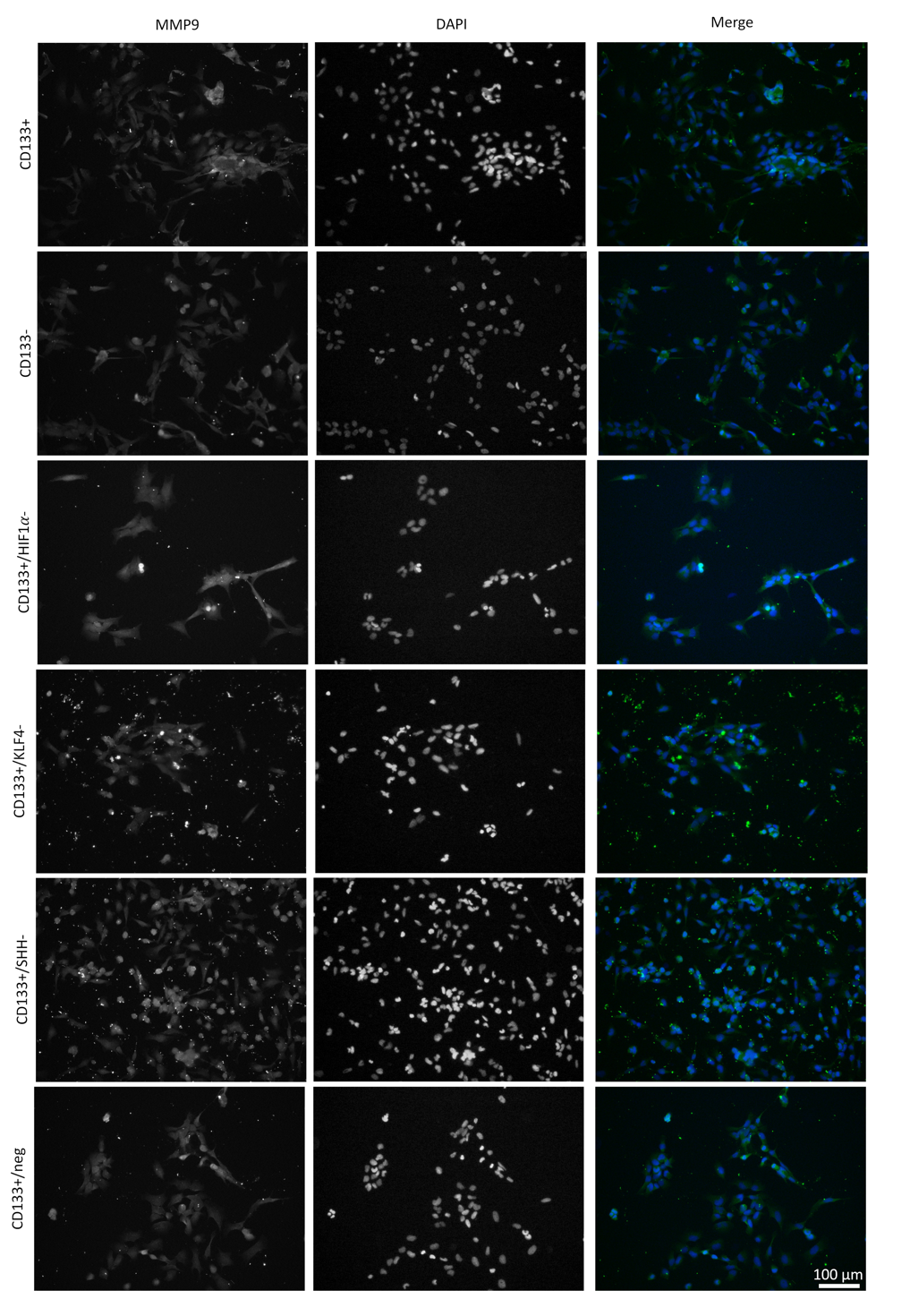
**

**Figure S10:** Representative images of MMP protein staining in grayscale, with DAPI staining indicating nuclei. In merged images, MMP9 is green and nuclei areblue. All experimental groups show comparable MMP9 protein intensity.

**
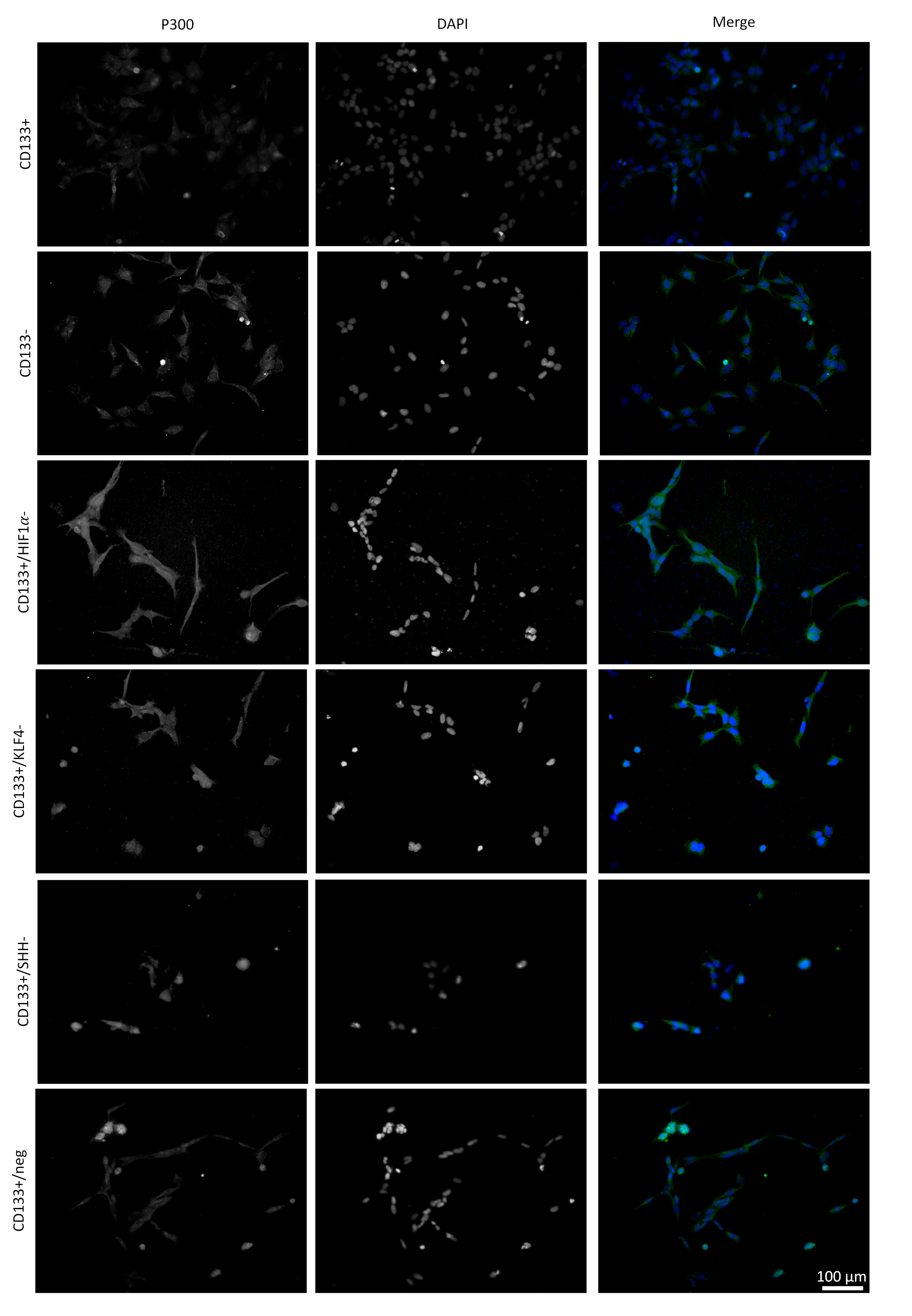
**

**Figure S11:** Representative images of P300 protein staining in grayscale, with DAPI staining indicating nuclei. In merged images, P300 is green and nuclei are blue. After treatment with all three siRNA separately, P300 expression increases.

**
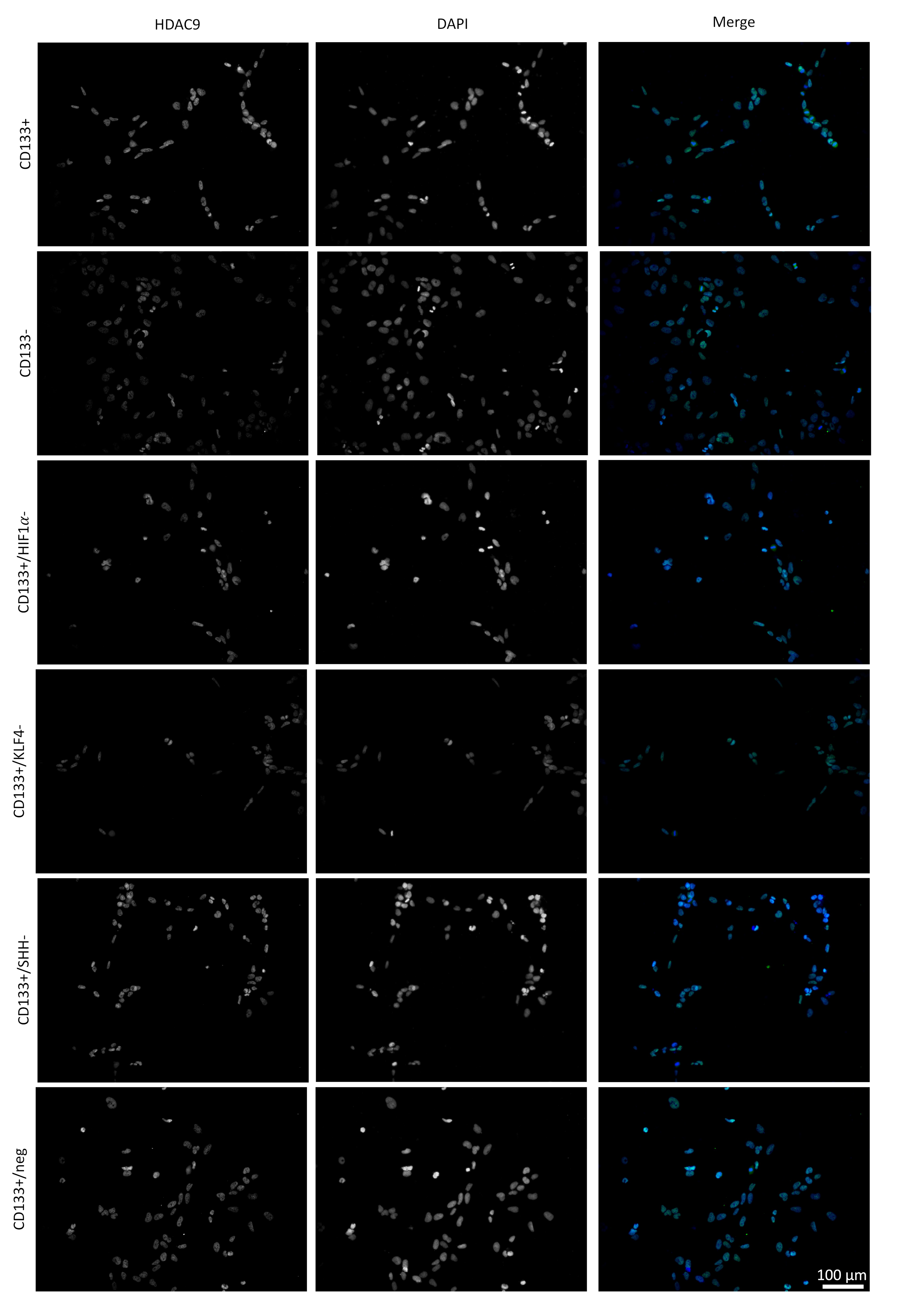
**

**Figure S12:** Representative images of HDAC9 protein staining in grayscale, with DAPI staining indicating nuclei. In merged images, HDAC9 is green and nuclei are blue. The CD133+ group exhibits higher HDAC9 expression compared to CD133- cell group. After KLF4 silencing, the expression level decreases.
